# DNA Repair Genes as Drug Candidates for Early Breast Cancer Onset in Latin America: A Systematic Review

**DOI:** 10.3390/ijms222313030

**Published:** 2021-12-02

**Authors:** Laura Keren Urbina-Jara, Emmanuel Martinez-Ledesma, Augusto Rojas-Martinez, Francisco Ricardo Rodriguez-Recio, Rocio Ortiz-Lopez

**Affiliations:** Tecnologico de Monterrey, Escuela de Medicina y Ciencias de la Salud, Monterrey 64710, Mexico; A00823119@itesm.mx (L.K.U.-J.); juanemmanuel@tec.mx (E.M.-L.); augusto.rojasmtz@tec.mx (A.R.-M.); franciscordzrecio97@gmail.com (F.R.R.-R.)

**Keywords:** DNA repair genes, breast cancer in young women, breast cancer datasets, cell lines, therapy

## Abstract

The prevalence of breast cancer in young women (YWBC) has increased alarmingly. Significant efforts are being made to elucidate the biological mechanisms concerning the development, prognosis, and pathological response in early-onset breast cancer (BC) patients. Dysfunctional DNA repair proteins are implied in BC predisposition, progression, and therapy response, underscoring the need for further analyses on DNA repair genes. Public databases of large patient datasets such as METABRIC, TCGA, COSMIC, and cancer cell lines allow the identification of variants in DNA repair genes and possible precision drug candidates. This study aimed at identifying variants and drug candidates that may benefit Latin American (LA) YWBC. We analyzed pathogenic variants in 90 genes involved in DNA repair in public BC datasets from METABRIC, TCGA, COSMIC, CCLE, and COSMIC Cell Lines Project. Results showed that reported DNA repair germline variants in the LA dataset are underrepresented in large databases, in contrast to other populations. Additionally, only six gene repair variants in women under 50 years old from the study population were reported in BC cell lines. Therefore, there is a need for new approaches to study DNA repair variants reported in young women from LA.

## 1. Introduction

Breast cancer (BC) is one of the main causes of death in women around the world [1], despite vast efforts to improve this outcome. Globally, an increase in BC is observed in women with ages between 35 and 54 years [2,3]. For example, in the United States, more than 12,000 women under 40 years old (y.o.) are diagnosed with BC each year [4]. Besides the rising BC incidence in young women <40 y.o. (YWBC) [5], more aggressive cancers have been observed in younger women [6]. At diagnosis, YWBC patients frequently present poorly differentiated and aggressive tumors characterized by lymph node invasion, deficiency of hormonal receptors, overexpression of human epidermal growth factor receptor type 2 (HER2), high proliferation rates, and advanced stage at diagnosis [7,8,9,10]. Likewise, higher occurrences of triple-negative breast cancer (TNBC) and basal-like types are observed in YWBC, both associated with worse prognosis [10]. Therefore, it is required to elucidate the underlying biological mechanisms implicated in YWBC development.

Studies have shown multiple altered variants in DNA repair genes implied in BC predisposition, development, and outcome [11,12,13,14], including BRCA and non-BRCA genes. Among these, pathogenic variants in the high penetrance *BRCA1/2* genes account for 50–60% and the remaining variants, to non-BRCA genes of moderate and low penetrance, including *ATM*, *PALB2*, *RAD51*, and *BARD1*, all involved in double-strand break repair pathways [15,16,17,18,19,20,21]. For this reason, it is relevant to elucidate the mechanisms of DNA repair genes in BC, using different approaches such as in silico, in vitro, and in vivo models.

A large amount of basic knowledge on BC is derived from in vivo and in vitro studies using BC cell lines, which provide a source of homogeneous materials that self-replicate. Therefore, cell lines are model systems useful to study cancer biology [22]. Moreover, the use of cancer cell lines is key to identify new drug targets and for the improvement of current therapeutic options focusing on drug sensitivity and resistance [23,24,25,26,27,28]. Hence, it is essential to obtain information about the genomic context of each cell line model to draw reliable conclusions on drug sensitivity in cell lines resembling the molecular characteristics of YWBC [29].

The need for reproducibility in clinical research brought a plethora of publicly available databases. Databases for cancer studies provide a trustworthy and useful resource to perform different analyses due to their large compilation of BC cases, including clinical and genomic data [30]. A benefit of database analysis is the discovery of elements obtained from the collection of samples of a specific condition to guide functional in vitro studies. In this way, we can test causal hypotheses and search for new therapeutic agents.

Although there are abundant clinical studies on BC, there is low representativeness of minorities in clinical databases [31]. While there are several reports of germline and somatic variants in BC from Latin America (LA), they are scarce compared to other populations. For this reason, this study aimed at providing preliminary information to address the lack of data regarding YWBC in our region. For this, we focus on contrasting DNA repair variants for BC reported in The Cancer Genome Atlas (TCGA) and the Molecular Taxonomy of Breast Cancer International Consortium (METABRIC) with DNA repair variants reported for BC in our subcontinent [32]. In addition, we searched for these LA variants in public repositories of drug response cancer cell lines to evaluate drug susceptibility.

## 2. Results

### 2.1. Tumor Samples Data for YWBC

Table 1 describes the clinical information from METABRIC and TCGA databases and shows distinctions regarding YWBC. In METABRIC, the youngest reported patient was a 21.9 y.o. woman with mixed ductal and lobular BC, HER2+, and known somatic variants in *TP53*, *NCOA3*, *MUC16*, and *AHNAK* genes (sample ID, MB-3467). In addition, the METABRIC sample MTS-T1284 (47 y.o., ER+, ductal BC) presented variants in *APC*, *ATR*, *BRCA1,* and *FANCA* genes. On the other hand, from the 292 samples aged <50 years in TCGA, only 11 (3.8%) samples were described as Hispanic-Latino. The mean age at diagnosis for this group was 42.4 years, the mean overall survival (OS) was 45 months, while the shortest and longest reported OS was 14.6 and 96 months, respectively.

Information concerning DNA repair variants characteristics from METABRIC and TCGA samples <50 y.o. are presented in Table 2. Interestedly, the Hispanic-Latino sample TCGA-EW-A2FV-01 presented variants in 23 DNA repair genes (*APEX1*, *ATM*, *CCNB2*, *CHEK1*, *CHEK2*, *FANCA*, *FANCD2*, *MSH3*, *PARP9*, *PMS2*, *POLD1*, *POLE*, *RAD50*, *RECQL4*, *RECQL5*, *REV3L*, *RIF1*, *RPA2*, *RPA4*, *SMC2*, *SMC3*, *TOP2A*, and *WRN*).

**Figure 1:**
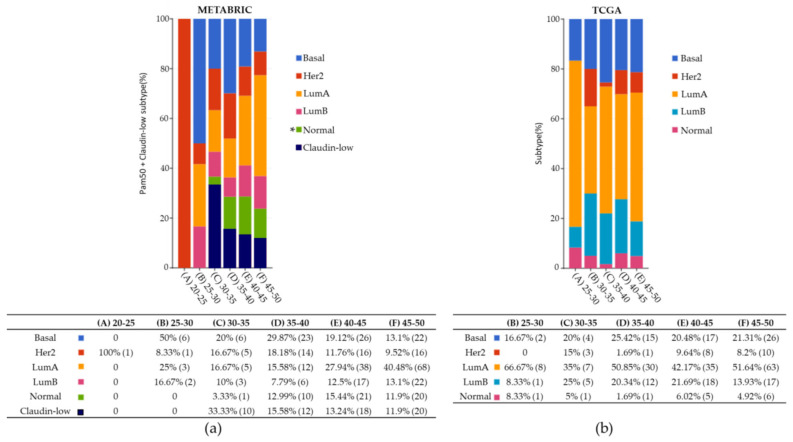
METABRIC and TCGA under 50 y.o. BC subtype. (**a**) METABRIC dataset divided into six different age groups. Chi-squared test *p*-value 5.705e-4. (**b**) TCGA dataset consisted of five age groups. Chi-squared test *p*-value 0.616. The number of samples is indicated in parentheses. * Normal-like is similar to luminal A, PR and/or ER positive, HER2 negative, and low Ki-67 levels.

Figure 1 shows the percentages of tumor subtypes observed in METABRIC and TCGA for BC samples <50 y.o. METABRIC dataset subtype classification includes Claudine-low subtype in contrast to TCGA. In METABRIC, the only patient in the age group 20–25 years had a HER2 subtype. The most common subtypes by age group were the following: basal and luminal A subtypes in the 25–30 years (50 and 25%, respectively), claudin-low and basal subtypes in the 30–35 years (33.3 and 20%, respectively), basal subtype in the 35–40 years (30%), and luminal A subtype in the 40–45 and the 45–50 years (27.9 and 40.5%, respectively) (Figure 1a). We performed a Chi-squared test to assess differences among the six age groups, mainly between the groups of 20–35 y.o versus 35–50 y.o. groups, observing basal and luminal A breast cancer subtypes as predominant. Interestingly but worrisome, the basal subtype was more frequent for age groups below 40 years.

In TCGA (Figure 1b), the luminal A subtype was the highest reported for all age groups (from 35% in the 30–35 years group up to 66.7% in the 25–30 years group). Luminal B subtype was present in the 30–35 years group (25%) and 40–45 years group (21.7%). Again, the basal subtype was prevalent in young age groups: the 25–30 years group (16.7%), 35–40 years group (25.4%), 40–45 years group (20.5%), and 45–50 years group (21.31%).

**Figure 2:**
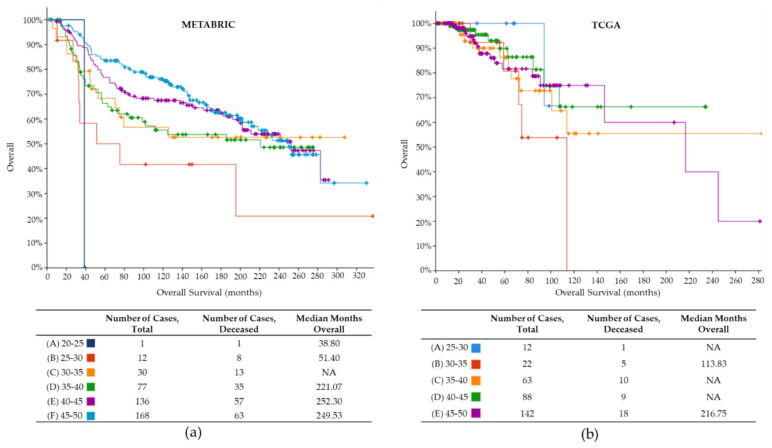
Overall survival METABRIC and TCGA under 50 y.o. The overall survival for six different age groups of women is represented in months. (**a**) METABRIC. The group of 30–35 y.o. in orange displays better overall survival (over 55% for 300 months), while the only case in the 20–25 years group shows the worst survival. The 45–50 years group concentrates more BC cases. Log-rank test *p*-value 0.0252. (**b**) TCGA. The 30–35 years group shows the worst overall survival, contrary to the METABRIC dataset. The best overall survival is observed for the 40–45 years group (over 60% survival at 220 months). Log-rank test *p*-value 0.454. NA, not available.

We analyzed the overall survival in the METABRIC and TCGA data for women <50 years for the 6 and 5 different age groups, respectively (Figure 2). For METABRIC, the lowest median overall survivals were 38.8 and 51.40 months in the groups 20–25 and 25–30 years, respectively. The 45–50 years group presented the longest median survival and had the highest number of patients. (Figure 2a). For the TCGA dataset, the 30–35 years groups had the worst overall survival, while the 40–45 years had the best (Figure 2b) [33,34]. This highlights the importance of a timely diagnosis of BC for young patients. Figure S1 presents survival analyses with BC subtype for both METABRIC and TCGA.

**Figure 3:**
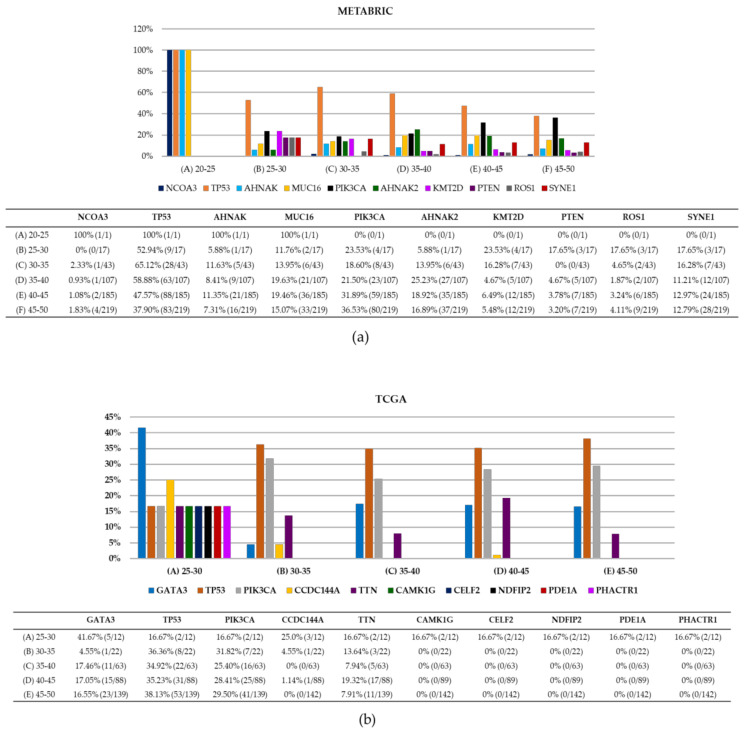
METABRIC and TCGA under 50 y.o. 10 most frequently mutated genes. (**a**) The METABRIC dataset is divided into six different age groups. Pathogenic variants in *TP53*, *PIK3CA*, and *MUC16* are frequently observed in all group ages. Frequencies of pathogenic variants for each gene group are illustrated. (**b**) The TCGA dataset consisted of five age groups. Pathogenic variants in *TP53*, *PIK3CA,* and *GATA3* are moderately prevalent in women <50 y.o. Five genes such as *CAMK1G*, *CELF2*, *NDFIP2*, *PDE1A*, and *PHACTR1* were observed in two patients only (16.67%) from the 25–30 age group.

Furthermore, we searched for pathogenic variants in the METABRIC and TCGA datasets for the same established age groups. In Figure 3, the 10 most frequent altered genes in YWBC are presented by age groups for both METABRIC and TCGA data. In METABRIC, pathogenic variants in the *TP53* gene were observed in all groups, followed by variants of the *PIK3CA* gene. The sample of the only patient in the 20–25 years group had pathogenic variants in *NCOA3*, *TP53*, *AHNAK*, and *MUC16* (Figure 3a). The TCGA dataset, in general, presented different results for the most frequently mutated genes in women <50 y.o. like the frequency of *CAMK1G*, *CELF2*, and *NDFIP2* in women aged 35–50 y.o., in contrast to METABRIC data. Some similarities to METABRIC data include *TP53* and *PIK3CA* genes as the most altered in the five age groups. *GATA3* was present in all groups (Figure 3b) [33,34].

**Figure 4:**
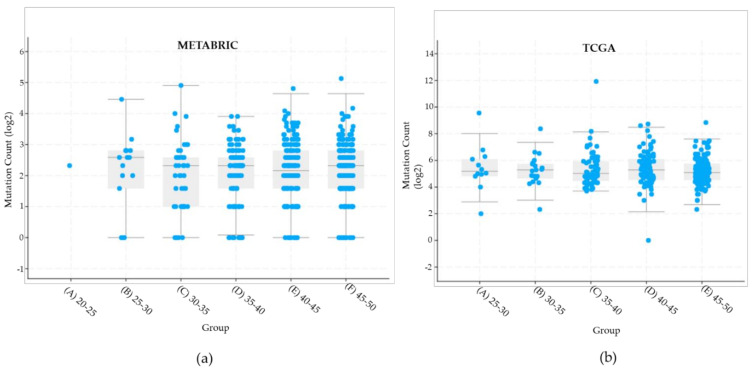
METABRIC and TCGA mutation load in patients with breast cancer under 50 y.o. (**a**) METABRIC dataset divided in six different age groups. Derived from Kruskal–Wallis test, *p*-value 0.910. (**b**) TCGA dataset consisted of five age groups. Derived from Kruskal–Wallis test, *p*-value 0.830. In general, a higher mutation count was observed in TCGA than METABRIC samples.

Moreover, we collected mutation load in these databases according to different age groups (Figure 4). We specify that the numbers mentioned in this paragraph are expressed in log2 as displayed in Figure 4. For METABRIC, the median mutation count was 2.58 and maximum 4.45 for the 25–30 y.o. group. In the following group of 30–35-year-olds, the maximum mutation count was 4.9 and a reported median of 2.32. The maximum count for the group of 35–40 y.o. was 3.9 and the median of 2.32. Likewise, a high mutation count of 4.64 was observed in the 40–45 y.o. group with a median of 2.12. Lastly, for the 45–50 y.o. group median mutation count was 2.32, and the maximum reported of 4.64 (Figure 4a). For the TCGA dataset, five age groups were analyzed. In the 25-30 y.o. a median 5.18 mutation count was reported with a maximum of 8.0. A median of 5.28 was observed in the 30–35 age group with a maximum of 7.35. For the 35–40 y.o group a maximum mutation count of 8.14 was reported with a median of 5.02. Moreover, a maximum of 8.48 and a median of 5.38 were observed in the 40–45 age group. Lastly, in the 45–50 y.o. group a maximum mutation count of 7.6 and a median of 5.09 was reported (Figure 4b). Curiously, the sample TCGA-EW-A2FV from a 39 y.o. woman has a mutation count of 12. Thus, a higher mutation load was observed in TCGA than METABRIC samples. This could be due to differences in screened populations, TCGA included women from more diverse ethnicities in contrast with the METABRIC study [35].

### 2.2. BC Cell Line Variants and DNA Repair Genes

**Figure 5:**
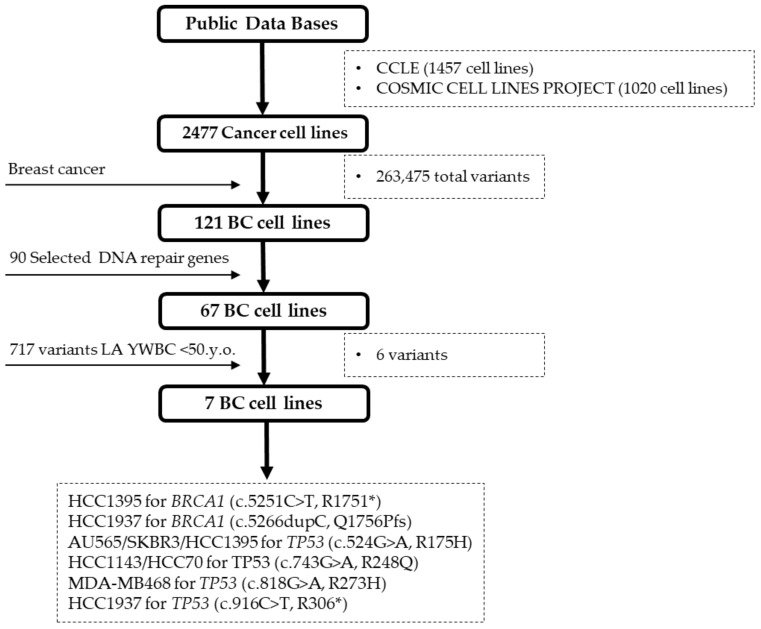
BC cell lines variants and DNA repair genes. CCLE and COSMIC Cell Lines Project databases were analyzed and a total of 2477 cancer cell lines were retrieved. Sixty-seven cell lines corresponded to BC. Cell lines were filtered for 90 DNA repair variants and 717 LA reported variants resulting in the selection of seven BC cell lines: AU565 (c.524G > A, *TP53*), HCC1143 (c.743G > A, *TP53*), HCC1395 (c.5251C > T, *BRCA1*; c.524G > A, *TP53*), HCC1937 (c.916C > T, *TP53*; c.5266dupC, *BRCA1*), HCC70 (c.743G > A, *TP53*), MDA-MB648 (c.818G > A, *TP53*), and SKBR3 (c.524G > A, TP53). * Truncating variant.

**Figure 6:**
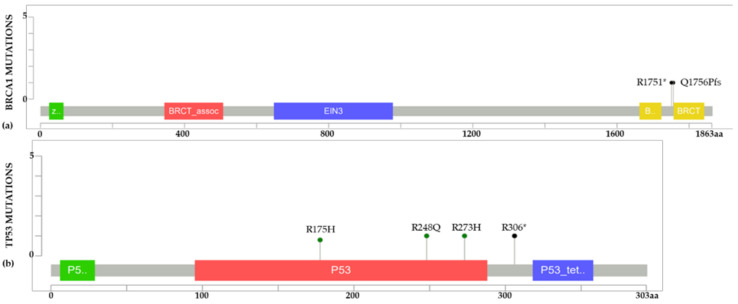
*BRCA1* and *TP53* LA variants in BC cell lines. (**a**) *BRCA1* nonsense variant R1751* (c.5251C > T) and frameshift variant Q1756Pfs (c.5266dupC) are displayed. (**b**) TP53 missense variant R248Q (c.743G > A), missense variant R175H (c.524G > A), missense variant R273H (c.818G > A), and nonsense variant R306* (c.916C > T) are represented. * Truncating variant.

For the analysis of cell line variants harboring mutated DNA repair genes two public databases were reviewed, CCLE and COSMIC Cell Lines Project. A total of 2477 cancer cell lines were retrieved, 1457 from CCLE and 1020 from COSMIC Cell Lines Project. Data was filtered for BC and DNA repair genes resulting in 67 BC cell lines for further analysis. Information regarding the analyzed BC cell lines is shown in Table S3. Cell lines were compared with our database of LA variants in DNA repair genes [32]. The final selection of cell lines consisted of seven BC cell lines: AU565, HCC1143, HCC1395, HCC1937, HCC70, MDA-MB468, and SKBR3, all carrying variants in *BRCA1* and *TP53* genes, as shown in Figure 5. Information on onset age, country, and variant was available for all the entries. Contributing countries for this dataset are limited to Brazil, Argentina, and Uruguay. Variants in *BRCA1* and *TP53* observed in BC cell lines are represented in the LA dataset (Figure 6) [33,34]. Two variants are displayed for *BRCA1*, c.5251C > T (R1751*) a nonsense variant, and c.5266dupC (Q1756Pfs) a frameshift variant. *TP53* presents three different variants, the missense variant c.743G > A (R248Q), the missense variant c.818G > A (R273H), and the nonsense variant c.916C > T (R306*). Comparing the 717 variants for 15 DNA repair genes that were reported in women under 50 y.o. with BC in Latin America, it can be inferred that the presence of these variants in BC lines is low. Therefore, only six variants for two genes (*BRCA1* and *TP53*) were observed in seven cell lines of a total of 121 BC cell lines analyzed. This analysis reflects the low representation of gene variants in BC lines besides *BRCA1*/2 and *TP53* in LA women <50 y.o. Information concerning the biological effects and clinical implications of each variant is described in Table S1.

### 2.3. Drug Sensitivity and Cell Lines with Altered Repair Mechanisms

**Figure 7:**
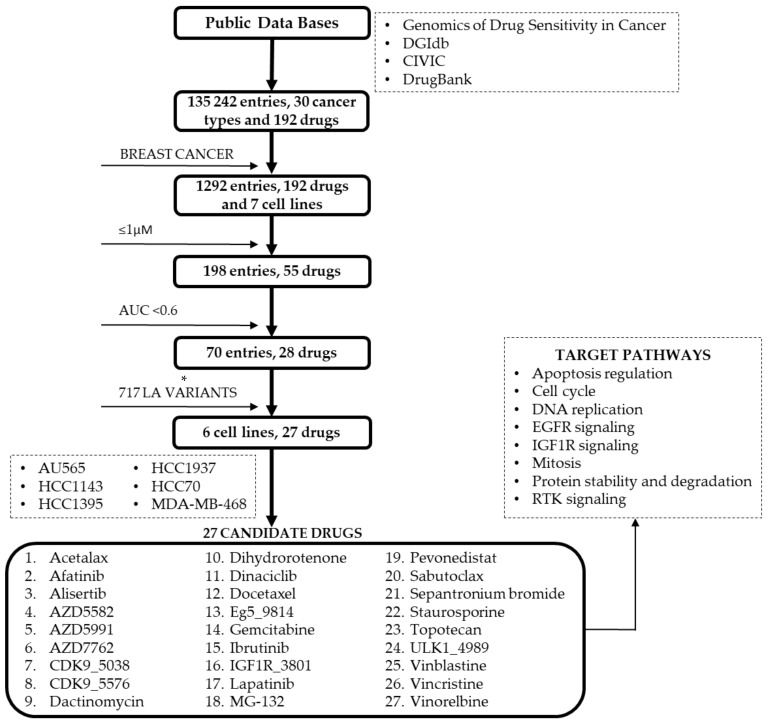
BC cell lines and variants in DNA repair genes. Four different databases were searched for this analysis obtaining information regarding 30 cancer types and 192 drugs. The BC filter data reduced 1292 entries to seven BC cell lines. Considering a concentration ≤1 μM for all drugs and an AUC <0.6 the search was narrowed to 70 entries and 28 drugs. Only six BC cell lines were selected after filtering cell lines carrying LA variants (AU565, HCC1143, HCC1395, HCC1937, HCC70, and MDA-MB-468) and 27 candidate drugs. Pathways impacted by these drugs include apoptosis regulation, cell cycle, DNA replication, EGFR signaling, IGF1R signaling, mitosis, protein stability and degradation, and RTK signaling. * Variants selected from our previous study [32].

For this analysis, the GDSC2 dataset from the Genomics of Drug Sensitivity in Cancer repository was evaluated. A total of 30 cancer types, 192 drugs, and 135,242 entries were retrieved. No PARP inhibitors were tested in these drug databases for the selected cell lines. Later, different filters were applied to obtain specific information. First, the BC filter displayed seven different BC cell lines. For this analysis, the concentration range selected was ≤1 μM for all drugs. Followed by a selection of drugs that displayed an area under the curve (AUC) lower than 0.6. With this information, LA variants from young women previously selected in this study were compared and drugs with the best performance in cell line assays were retrieved. Six BC cell lines were retrieved: AU565, HCC1143, HCC1395, HCC1937, HCC70, and MDA-MB-468. A total of 27 candidate drugs were collected from this analysis, including drugs impacting different pathways such as apoptosis regulation, cell cycle, DNA replication, IGF1R signaling, mitosis, protein stability and degradation, and receptor tyrosine kinase (RTK) signaling (Figure 7).

**Figure 8:**
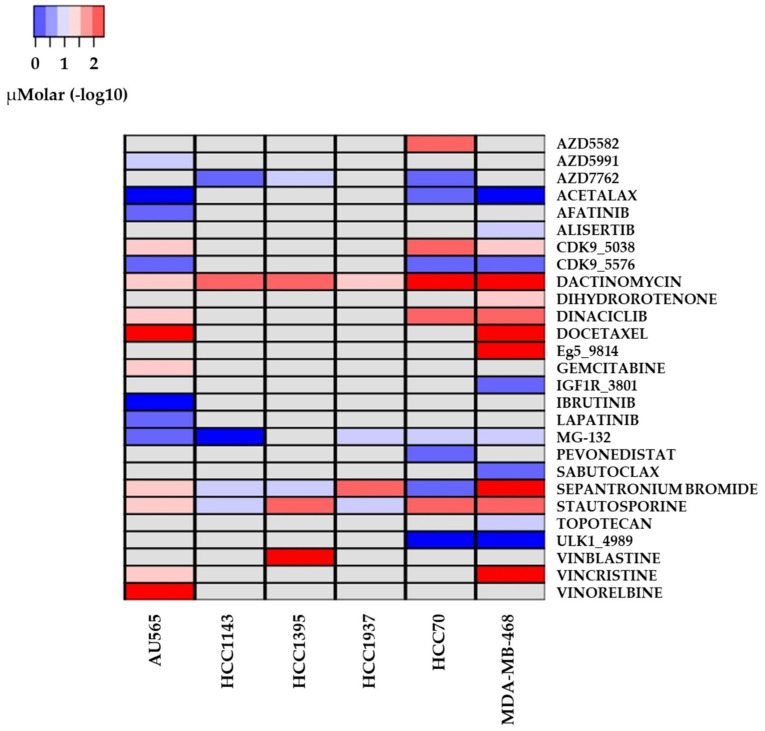
The 27 candidate drug μM concentration for fitted IC50, considering the parameters previously described in Figure 7, concentration ≤1 μM and AUC < 0.6. Each of the 27 candidate drugs with best results according to previously mentioned criteria are shown in this image. Drugs displaying the lowest concentrations include dactinomycin, docetaxel, and vincristine. On the other hand, drugs with highest concentrations are acetalax, ibrutinib, and ULK1_4989. Drug concentrations (μM) are displayed in blue and red shades. Grey spots represent data not available.

Further, Figure 8 shows the fitted IC50 μM concentration of each of the 27 candidate drugs from this search. Drugs such as dactinomycin, docetaxel, and vinorelbine have the lowest μM concentration. On the other hand, drugs like ULK1_4989 and acetalax are the ones displaying the highest μM concentration. Table S4 presents all drug information for these analyses.

Herein Table 3 describes the 27 candidate drugs with their putative target and the pathway in which each drug interacts. In addition, BC cell lines reporting these drugs are displayed.

## 3. Discussion

### 3.1. Importance of Combining Database Information and Cell Lines

Databases serve as preliminary approaches in preclinical in vitro and in vivo assays to address inquiries regarding pathogenicity and drug candidates. For this study, the TCGA and METABRIC databases were analyzed to identify frequent variants in DNA repair genes in YWBC. In addition, using these tools we were able to compare reported variants in different age groups from LA countries.

On the other hand, in vitro studies with cancer cell lines remain relevant for advancing clinical cancer research because they resemble molecular characteristics and allow analyses on therapy response in drug evaluation. In addition, they are an important tool to perform large-scale drug sensitivity assays and interactions among drugs and genes, which are fundamental in Precision Medicine. These studies are unbiased for exploring alleged factors of drug sensitivity [23]. Cancer therapy approaches are being improved by new technologies capturing the clinical and molecular profiles of a patient and allow statistical assessment against impressive sets of information stored in public databases [28].

### 3.2. Analysis of Pathogenic Variants of YWBC in LA and Public Databases

The analysis performed in the METABRIC and TCGA databases shows that there is a significant presence of young women <50 years of age in both databases. It is observed that different subtypes of BC predominate in each age group, such as basal and luminal A, in patients in the 25–30-year-old group. Remarkably, survival analysis shows that young women between the ages of 25 and 35 have the lowest survival rates. This group includes a 21-year-old woman registered in the METABRIC database. DNA repair genes that are frequently altered in both METABRIC and TCGA include *TP53*, *ATR*, and *FANCA*.

As expected, mutational load analysis shows accumulation of mutations as a function of aging. Unfortunately, there is an underrepresentation of YWBC from LA in the METABRIC (no patients) and TCGA (11 patients) studies to corroborate this assertion. According to Wojtyla et al., BC death rates are not decreasing in LA countries. For women aged 20–49 years, low mortality rates are observed only in Chile and Cuba, while an increase in mortality is observed in the rest of the LA countries [36]. Although the incidence of BC is higher in the US and European populations, the mortality–incidence ratio is higher for LA, as described by Dutil et al. [37]

Another observation from this study is that the mutational profile of many populations from LA is limited, as concluded by Ren at al. [38]. We observed that the DNA repair gene variants reported in LA are not well represented in commercial BC cell lines, so this issue needs to be addressed. Furthermore, variants without defined pathogenicity abound in patients from the region, and since these variants are not represented in BC cell lines, the functional analysis of DNA repair pathways is troublesome. Some methodologies, such as CRISPR/Cas9 edition [39,40], or the analysis of established patient-derived cell lines, can be used to perform studies to define the pathogenicity of these particular variants.

### 3.3. Drugs with Reported Sensitivity in BC Cell Lines

In this study, 27 drugs were identified with effect in six BC cell lines (AU565, HCC1143, HCC1395, HCC1937, HCC70, and MDA-MB-468). In addition, according to CIVIC database there are no FDA-approved or NCCN-compendium register treatments especially for patients that carry some of the LA reported variants such as R248Q variant for *TP53*, since this variant has been correlated with worse overall survival in BC patients in contrast to wild-type [41,42].

Some drugs are being tested in clinical trials, others are still being assessed in preclinical models, particularly in cultured lines. Information for some drugs was either scarce or null. Among these, sepantronium bromide (YM-155) targets the survivin protein inducing anti-apoptotic capacity. Preclinical and phase I clinical trials reported promising results, nonetheless, a poor performance was observed in phase II clinical trials for different cancer types. Wani et al. developed a BC cell line (MCF-7) expressing resistance to YM-155 and observed that continuous treatment with YM155 resulted in a low expression of survivin. This compound confers its chemotherapeutic effect by causing oxidative stress-mediated DNA damage. Therefore, DNA damage-response pathway proteins will be appropriate predictive biomarkers of YM155 response in these cells [43]. Mazzio et al. treated MDA-MB-231 BC cells with YM155 and observed changes in survivin mRNA and protein levels. Findings suggest that YM-155 inactivates replication-dependent DNA repair systems. They reported upregulation of *SIK1* and *FOSB* (tumor suppressors), *KDM6B* (histone methylation), and *NOCT*, *PER*, *BHLHe40*, and *NFIL* (circadian rhythm). Conversely, downregulation of *GUSBP3* (glucuronidase), some micro-RNAs, and DNA damage repair players like *CENPI*, *POLQ*, *RAD54B* was also observed. The most affected pathways were *ATM/FANC* (*FANC2*, *FANCI*, *BRCA1*, *BRCA2*, *RAD51*, *PALB2*) and *ATR* [44]. 

Moreover, for the acetalax drug, Rajapaske et al. propose repositioning acetalax as an anticancer drug candidate for triple-negative BC based on their cytotoxic and antiproliferative activities in BC cell lines [45]. Similar findings were reported by Morrison and collaborators [46].

A study conducted by Hennessy et al. with AZD5582 showed apoptosis induction in BC MDA-MB-231 cell line at subnanomolar concentrations characterized by cIAP1 degradation. These observations were corroborated in xenograft-bearing mice implanted with MDA-MB-231, in which tumor regression was achieved and molecular analyses confirmed the degradation of cIAP1 this compound is being considered as a candidate for clinical trials [47]. Similarly, Polanski et al. conducted a combined screening employing 31 BC cell lines and demonstrated synergy between TRAIL and AZD5582 in approximately 30% of tested cell lines. This effect was correlated with sensitivity to TRAIL, but not to AZD5582 as a single agent. Most of AZD5582 + TRAIL-resistant BC cell lines preserving a functional death route were sensitive to AZD5582 + TNF*α* combination treatment [48].

AZD7762 (DN10764), a selective inhibitor of checkpoint kinases 1 and 2, has been reported as useful to suppress BC metastasis. Park et al. observed that this compound inhibited cell proliferation and GAS6-mediated AXL signaling, resulting in reduced invasion and migration capabilities. It has been shown that this drug promotes caspase 3/7 apoptosis in BC cells. Similar results were observed in in vivo metastasis models. This study suggests that combined therapy strategies targeting AXL, like AZD7763, could improve responses in drug-resistant solid tumors where AXL is involved, such as NSCLC, BC, ovarian cancer, and others [49]. Moreover, Min et al. demonstrated synergism between gemcitabine (GEM) and AZD7762 in a TNBC cell line [50].

Alsamman et al. examined an approach to overcome cisplatin resistance using staurosporine in breast, colon, and ovarian cancer cell lines by evaluating proliferation, morphology, and p62 levels after one of three treatments (cisplatin, staurosporine, or both combined). Results showed an elevation of p62 levels after cisplatin treatment. On the contrary, a reduction of p62 level was reported after staurosporine treatment. These results propose that cancer cells could be sensitized to cisplatin using staurosporine by downregulation of p62 [51].

Some combination therapies are studied and used in selected cancer types such as TNBC and NSCLC. These therapies show benefits and promising results, yet the disadvantages of treatments like doxorubicin against TNBC and NSCLC are toxicity and resistance. Studies conducted by Ghosh et al. explored a combined therapy with doxorubicin and vincristine for these cancer types, optimizing a single PEGylated liposomal formulation. A significant reduction (*p* < 0.05) of IC50 and cell viability of cell lines A549 (NSCLC) and MDA-MB-231 (BC) was observed compared with single drug treatments. This observation was corroborated in in vivo studies showing enhanced tumor reduction after vincristine plus doxorubicin therapy [52,53].

According to Falchook et al., alisertib (MLN8237), a selective Aurora A Kinase (AAK) inhibitor, shows good antitumor and AAK inhibitor activity in xenograft models consisting of different tumor types. This compound exhibited antitumor activity at an oral dose of 40 mg twice daily plus weekly paclitaxel 60 mg/m^2^ in patients with ovarian cancer. Therefore, future studies of alisertib in combination with taxanes and paclitaxel are expected [54]. Alisertib did not perform as expected as a single agent in phase III trials, but there is a clinical trial phase I (NCT02219789) evaluating its combination with fulvestrant [55]. It has been suggested that the combination of Alisterib with other agents may reduce the toxicity of anticancer drugs, increasing their anticancer effects [56].

In a study conducted by Li et al., docetaxel was evaluated in combination with lobaplatin versus docetaxel combined with gemcitabine for treatment in 26 patients with recurrent metastatic BC for each treatment group. Lobaplatin is a dual inhibitor targeting EGFR and HER2 which do not show cross-resistance with cisplatin and this combination is effective in BC. Gemcitabine has also shown certain effects in recurrent metastatic BC. Complete remission was observed in five patients (11.6%), three patients with docetaxel and lobaplatin and two patients with combined therapy of docetaxel and gemcitabine, and partial response in 16 patients (37.2%). Reported response rates of the groups were comparable (47.6%, 50.0%). Median survival times after relapse and metastasis of the docetaxel and gemcitabine group were 25 months, whereas the docetaxel and lobaplatin group reported 18 months. Similar results were observed for median progression-free survival after relapse and metastasis. Therefore, researchers concluded that these combinations are effective and tolerable for advanced BC [57]. Similarly, alternative combination therapy for HER2-negative BC of docetaxel and cyclophosphamide (TC) was assessed by Caparica and collaborators in a meta-analysis, compared six cycles of this combined therapy versus an anthracycline and taxane-based regimen (A + T) in the adjuvant treatment of HER2-negative BC. As a result, they conclude that for adjuvant treatment in this subtype of BC, A + T treatment was associated with more toxicity risks and no clear survival benefit when compared with six cycles of TC. Therefore, they considered that A + T may be more beneficial for patients with higher risk, whereas for patients with lower risk TC combination therapy may be an effective and lower toxic alternative [58].

A study conducted by Lin et al. [59] in lung cancer, suggests that dactinomycin upregulates p53, and lung cancer cells (A549) result in growth suppression and apoptosis. Similarly, a study conducted by Das et al. reported this drug as a potential anti-BC agent using an in vivo-like 3D cell culture system for identification and validation of anti-cancer agents. Herein, it was shown that actinomycin D targets Sox-2, a stem cell transcription factor, and downregulates its expression resulting in a reduction of stem-cell population and stalling of the tumor progression initiation [60].

Sinha and collaborators evaluated topotecan effects in MCF-7 BC cells. The study reported that reactive free radical species are key players in cancer cell death. Topotecan significantly downregulates the estrogen receptor alpha (*ERα/ESR1*) and the *BCL2* genes [61]. In addition, studies such as that conducted by Guo et al. explore the combination of topotecan with other agents like daidzein, a phytoestrogen in vitro. Results showed strong synergistic effects on MCF7 cells by arresting the G2/M cell cycle phase and inducing apoptosis [62].

Hu et al. tested sabutoclax, a BCL-2 protein family antagonist, in two chemoresistant BC cell lines in vitro and in vivo, showing a significant cytotoxic effect and elimination of the cancer stem cell-like subpopulation. These findings suggest that sabutoclax partially overcomes drug resistance by inducing apoptosis mediated by inhibition of BCL-2 family proteins. These results stimulate further studies to explore the efficacy of sabutoclax alone or in combination in BC patients with nonresponsive chemotherapy [63].

Dinaciclib, a cyclin-dependent kinase (CDK) 1/2/5/9 inhibitor, has shown encouraging results in preclinical studies and is being evaluated in phase I clinical trials for BC treatment [64]. In a study conducted by Johnson et al., dinaciclib reported activity against CDK12, a regulator for transcription in HR, as well as previously mentioned CDKs. They observed that this drug reverses resistance to PARPi and eliminates HR repair in TNBC cells and xenografts derived from patients with mutated BRCA. Therefore, these results underline the importance of blocking HR repair for therapeutic purposes, encouraging combination therapies for TNBC such as dinaciclib as CDK12 and PARP inhibitors [65]. Similarly, in a study conducted by Zhu et al., dinaciclib was reported to restore sensitivity to cisplatin in cells with resistance to tamoxifen [66]. Dinaciblin is currently in phase III clinical trials. In addition, Nie et al. reported that inhibition of CDK2/EZH2 restores the expression of estrogen receptor alpha (ERα) leading to tamoxifen in vitro and in vivo sensitivity [67]. On the other hand, no information was found for drugs like AZD5991, CDK9_5038, CDK9_5576, Dihydrorotenone, Eg5_9814, IGF1R_3801, ULK1_4989, and Vinblastine for BC therapy.

Furthermore, one limitation of this study is that there are few studies reporting DNA repair variants in YWBC in LA countries, therefore variants representing this population are unknown. Another limitation of this study is that not all the variants reported are totally validated in public databases, some of them are reported as variants of unknown significance (VUS), hence, the effect of these variants needs to be studied. In addition, there are few BC cell lines that represent variants reported in LA.

## 4. Materials and Methods

**Figure 9:**
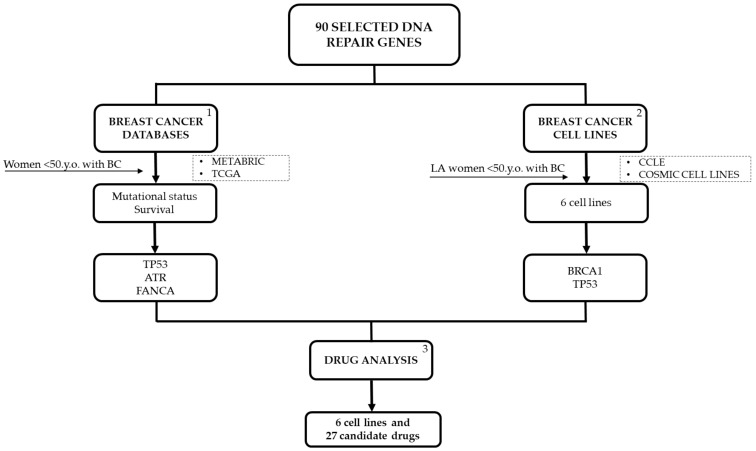
Flow chart followed in this study. (1) For further details refer to Figure 2; (2) for further details refer to Figure 5; (3) for further details refer to Figure 7. CCLE, Cancer Cell Line Encyclopedia; METABRIC, Molecular Taxonomy of Breast Cancer International Consortium; TCGA, The Cancer Genome Atlas.

For this study, 90 selected DNA repair genes were analyzed in BC databases such as TCGA, METABRIC, CCLE, and COSMIC Cell Lines. All this information was specific for women under 50 years old (y.o.), tumor sample data was analyzed for METABRIC and TCGA. For Latin American women, germline variants were considered. In addition, drug analyses were studied specifically for BC cell lines that represented DNA repair variants reported in LA YWBC (Figure 9).

### 4.1. DNA Repair Genes of Interest

For this study a selection of 90 genes were analyzed in different databases (Table 4). For this list, genes that participate in DNA repair pathways such as homologous recombination, non-homologous end joining, base excision repair, nucleotide excision repair and mismatch repair, were considered. In addition, this list includes genes that perform in the cell cycle and are involved in DNA repair mechanisms [13,21,68,69,70,71].

### 4.2. Young Women with BC Tumor Sample Data

Data was retrieved from TCGA and METABRIC from https://www.cbioportal.org/ accessed on 23 March 2021, selecting patients under 50 years old at diagnosis for further analysis. A total of 2509 samples were obtained from METABRIC, for the purpose of this study only samples of patients under 50 years were selected. A total of 567 samples from METABRIC were used to complete further analyses. From the TCGA database, a total of 1084 samples were retrieved. Considering the selection criteria of women under 50 years, 292 samples were chosen for further analyses. Later, a search for 90 DNA repair genes was made to obtain data of frequently altered genes and information concerning age at diagnosis and other clinical data such as BC type and survival from these samples.

### 4.3. BC Cell Lines Selection and Mutation Analysis for DNA Repair Genes

Data for a panel of 52 human BC cell lines was obtained from The Catalog of Somatic Mutations in Cancer (COSMIC) Cell Line Project database, and 69 BC cell lines from The Cancer Cell Line Encyclopedia (CCLE). In Table S3 a list of retrieved cell lines is noted along with BC subtypes [22,72,73,74,75,76,77] as well as reported mutations in DNA repair genes. Complete mutation data was obtained from https://cancer.sanger.ac.uk/cell_lines/download (last accessed on 21 November 2021), data file was filtered by cancer type, BC and analyzed. For CCLE, https://portals.broadinstitute.org/ccle (last accessed on 21 November 2021), the CCLE_DepMap_18q3_maf_20180718.txt/merged mutation calls (coding region, germline filtered) data was analyzed for this study. A total of 227,855 mutations were retrieved from COSMIC BC cell lines. Following, this data was filtered searching for the 90 DNA repair genes selected, to identify all the cell lines harboring mutations in one or more of these genes obtaining 2335 reported mutations in 52 BC cell lines for 84 DNA repair genes. In addition, for CCLE, a total of 35,590 mutations were obtained for BC cell lines. After selecting mutations for the 90 DNA repair genes 274 mutations remained for 74 genes in 65 cell lines. Furthermore, an analysis was performed to identify cell lines that presented frequent DNA repair variants observed in young women with BC. This selection will be useful to implement further in vitro analysis of candidate cell lines with altered DNA repair genes and evaluate drug sensitivity.

### 4.4. Drug Variant Sensitivity

Data from databases was analyzed aiming to spot drug sensitivity, half maximal inhibitory concentration (IC50), in BC cell lines with altered DNA repair genes. Information for drug–gene interaction was obtained from The Drug Interaction DataBase (DGIdb) (http://www.dgidb.org/search_interactions, last accessed on 21 November 2021) [30] and The Genomics of Drug Sensitivity in Cancer Project (https://www.cancerrxgene.org/, last accessed on 21 November 2021), a database to find drug response and genomic markers of sensitivity. In addition, Clinical Interpretation of Variants in Cancer (CIVIC) (https://civicdb.org/home, last accessed on 21 November 2021) and Drugbank (https://www.drugbank.ca/, last accessed on 21 November 2021) databases were used to gather supporting data for reported variants with drug sensitivity in previously mentioned databases. The Drugbank database is a bioinformatics resource that provides drug data combined with target information.

### 4.5. Reported DNA Repair Variants for YWBC from LA

**Figure 10:**
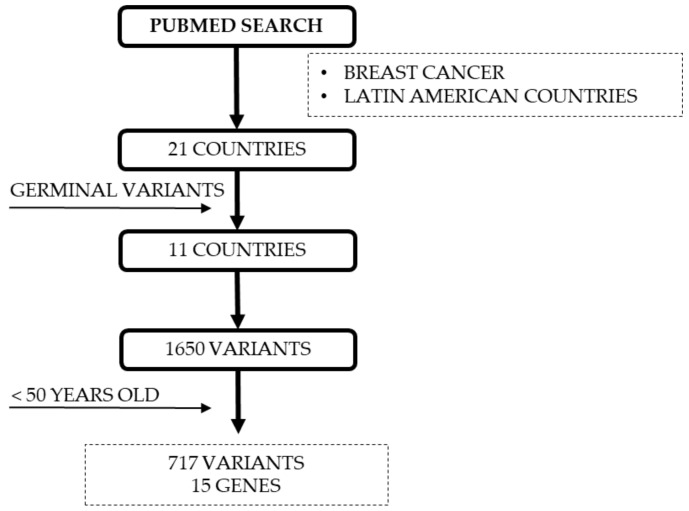
Variants selection for this study. Considering our previous study, a PUBMED search was performed for BC and LA countries. Herein, 11 out of 21 countries reported germline variants with a total of 1650 variants. After selecting only variants with reported age-onset under 50 years old, 717 variants remained for 15 genes. This database was used for this study.

A comparative analysis took place between frequent reported variants observed in YWBC from LA countries [32,78] and reported variants from databases such as TCGA, METABRIC, COSMIC, and CCLE. Variant selection for this study is shown in Figure 10. Table S1 contains all variants reported for DNA repair genes along with their effect reported by ClinVar, COSMIC, and PolyPhen. Information regarding BC subtype was available only for 20% of all the reported variants (Table S2).

## 5. Conclusions

There is vast information supporting the participation of DNA repair genes in BC and their possible implication in predisposition, development, outcome, and therapy response. Even though there is a wide range of publicly databases and studies addressing DNA repair variants, the representation for populations such as the Latin-American is minor and in contrast with other regions of the world reported data is limited. For the METABRIC and TCGA study there was an underrepresentation of YWBC from LA countries. Added to that, BC cell lines do not represent frequent DNA repair variants reported in countries from our region. Therefore, there is an opportunity area to address this matter.

The use of cell lines is fundamental for drug analysis, along with cell lines databases for preliminary studies. Herein, comparing LA-reported DNA repair variants from women <50 y.o. with BC cell lines resulted in a minor representation of these variants observing only seven BC cell lines for *BRCA1* and *TP53* genes. In addition, from the 15 DNA repair genes identified in young women <50 y.o. in LA, there are no BC cell lines that carry variants for 13 of those genes. Therefore, it represents a challenge to study these variants reported in the LA region and their research focusing on preliminary drug assays.

## 6. Take Home Messages

- Of our selection of 90 genes participating in DNA repair mechanisms, no representativeness of variants reported in LA was observed for YWBC <50 y.o., compared to variants from other populations.
- Cell line database analyses resulted in few variants representation of YWBC from LA countries in BC cell lines.
- More studies for LA population are needed to approach DNA repair genes and mechanisms for YWBC besides *BRCA1*, *BRCA2*, and *TP53* to generate cell lines that represent variants for this population.
- Our analyses resulted in different candidate drugs, nevertheless, not all of these drugs are validated in clinical practice.

## Figures and Tables

**Table 1:** Clinical characteristics of patients <50 y.o. from METABRIC and TCGA datasets.

Clinical Characteristics	METABRIC	TCGA
Total samples clinical data	567	292
Mean age at diagnosis (years)	42.4	42.4
Youngest age at diagnosis (years)	21.9	26.0
Mean OS (months)	134.1	40.4
Lowest OS (months)	1.4	0.0
Highest OS (months)	337	283
BC Subtype		
Lum A	22.2%	44.9%
Basal	14.6%	20.2%
Claudine-low	10.6%	0
HER2	9.3%	5.5%
Lum B	8.8%	16.8%
Normal	9.2%	4.1%
NA	25.3%	8.5%
Total samples	567	292

Lum A, luminal A; Lum B, luminal B; NA, not available; OS, overall survival.

**Table 2:** Features of the METABRIC and TCGA DNA repair variants.

	METABRIC	TCGA
Total samples (all ages)	2509 samples	1084 samples
Total variants all samples, all genes	17,272 variants	130,495 variants
Patients <50 years old		
Total samples	567 samples	292 samples
Total variants all genes	3839 variants	1693 variants
Top 5 mutated genes	*TP53*, *PIK3CA*, *MUC16*, *SYNEI1*, and *AHNAK2*	*TP53*, *PRKDC*, *ATM*, *BRCA2* and *BRCA1*
Reported DNA repair variants	420 variants	269 variants
Reported DNA repair genes	11 genes	90 genes
Samples with DNA repair variants	314 samples	122 samples
Top reported DNA repair genes	*TP53*, *ATR*, and *FANCA*	*TP53*, *ATM*, and *POLQ*
Gene with most variants	*TP53* (275 variants)	*TP53* (80 variants)
Most frequent variant	*TP53* p.R175H (12 samples)	*TP53* p.R175H (6 samples)
Sample with most reported DNA repair variants	MTS-T1284, 4 variants in *APC*, *ATR*, *BRCA1*, and *FANCA*	TCGA-EW-A2FV-01, 23 variants in 23 genes

**Table 3:** Drugs identified for treated BC cell lines with LA variants.

Drug Name	Cell Lines	Putative Target	Pathway Name
Acetalax	A565, HCC70, MDA-MB-468	-	Unclassified
Afatinib	A565	ERBB2, EGFR	EGFR signaling
Alisertib	MDA-MB-468	AURKA	Mitosis
AZD5582	HCC70	XIAP, cIAP	Apoptosis regulation
AZD5991	A565	MCL1	Apoptosis regulation
AZD7762	HCC1143, HCC1395, HCC70,	CHEK1, CHEK2	Cell cycle
CDK9_5038	A565, HCC70, MDA-MB-468	CDK9	Cell cycle
CDK9_5576	A565, HCC70, MDA-MB-468	CDK9	Cell cycle
Dactinomycin	A565, HCC1143, HCC1395, HCC1937, HCC70, MDA-MB-468	RNA polymerase	Other
Dihydrorotenone	MDA-MB-468	-	Unclassified
Dinaciclib	A565, HCC70, MDA-MB-468	CDK1, CDK2, CDK5, CDK9	Cell cycle
Docetaxel	A565, MDA-MB-468	Microtubule stabilizer	Mitosis
Eg5_9814	MDA-MB-468	KSP11	Other
Gemcitabine	A565	Pyrimidine antimetabolite	DNA replication
Ibrutinib	A565	BTK	Other, kinases
IGF1R_3801	MDA-MB-468	IGFR1	IGF1R signaling
Lapatinib	A565	EGFR, ERBB2	EGFR signaling
MG-132	A565, HCC1143, HCC1937, HCC70, MDA-MB-468	Proteasome, CAPN1	Protein stability and degradation
Pevonedistat	HCC70	NAE	Other
Sabutoclax	MDA-MB-468	BCL2, BCL-XL, BFL1, MCL1	Apoptosis regulation
Sepantronium bromide	HCC1143, HCC1395, HCC1937, HCC70, MDA-MB-468	BIRC5	Apoptosis regulation
Staurosporine	A565, HCC1143, HCC1937, HCC70, MDA-MB-468	Broad-spectrum kinase inhibitor	RTK signaling
Topotecan	MDA-MB-468	-	DNA replication
ULK1_4989	HCC70, MDA-MB-468	ULK1	Other, kinases
Vinblastine	HCC1395	Microtubule destabilizer	Mitosis
Vincristine	A565, MDA-MB-468	-	Mitosis
Vinorelbine	A565	Microtubule destabilizer	Mitosis

**Table 4:** Selection of DNA repair genes for this study.

APEX1	CHEK2	LIG4	POLD2	SSBP1
ATM	CTiP	MDC1	POLE	STK11
ATR	DNTT	MLH1	POLH	STRA13
BARD1	ERCC3	MLH3	POLQ	TIMELESS
BLM	ERCC6	MRE11A	PP4C	TOP2A
BRCA1	EXO1	MSH2	PRKDC	TOP3A
BRCA2	FAM175A	MSH3	RAD50	TOPBP1
BRIP1	FANCA	MSH6	RAD51	TP53
CCNA2	FANCB	MUTYH	RAD51C	TP53BP1
CCNB1	FANCC	NBN	RAD51D	TRIP13
CCNB2	FANCD2	NEIL2	RAD52	UBE2T
CDC25A	FANCI	NHEJ1	RECQL4	UIMC1
CDK1	FANCL	PALB2	REV3L	WRN
CDK12	FANCM	PARP1	RIF1	XPA
CDK4	GADD45B	PARP2	RPA1	XPC
CDKN2A	GEN1	PARPBP	RPA2	XRCC1
CENPS	H2AFX	PCNA	RRM2	XRCC5
CHEK1	HDAC2	PMS2	SMC1A	XRCC6

## Supplementary Materials

The following are available online at https://www.mdpi.com/article/10.3390/ijms222313030/s1.
