# Supplementary material for: DNA Repair Genes as Drug Candidates for Early Breast Cancer Onset in Latin America: A Systematic Review
Source: Int J Mol Sci. 2021 Dec 2;22(23):13030. doi: 10.3390/ijms222313030 (PMC8657579; doi:10.3390/ijms222313030)
Supplement: Supplementary file 1 [file ijms-22-13030-s001.zip › Supplementary Figure S1- proofread.pdf]

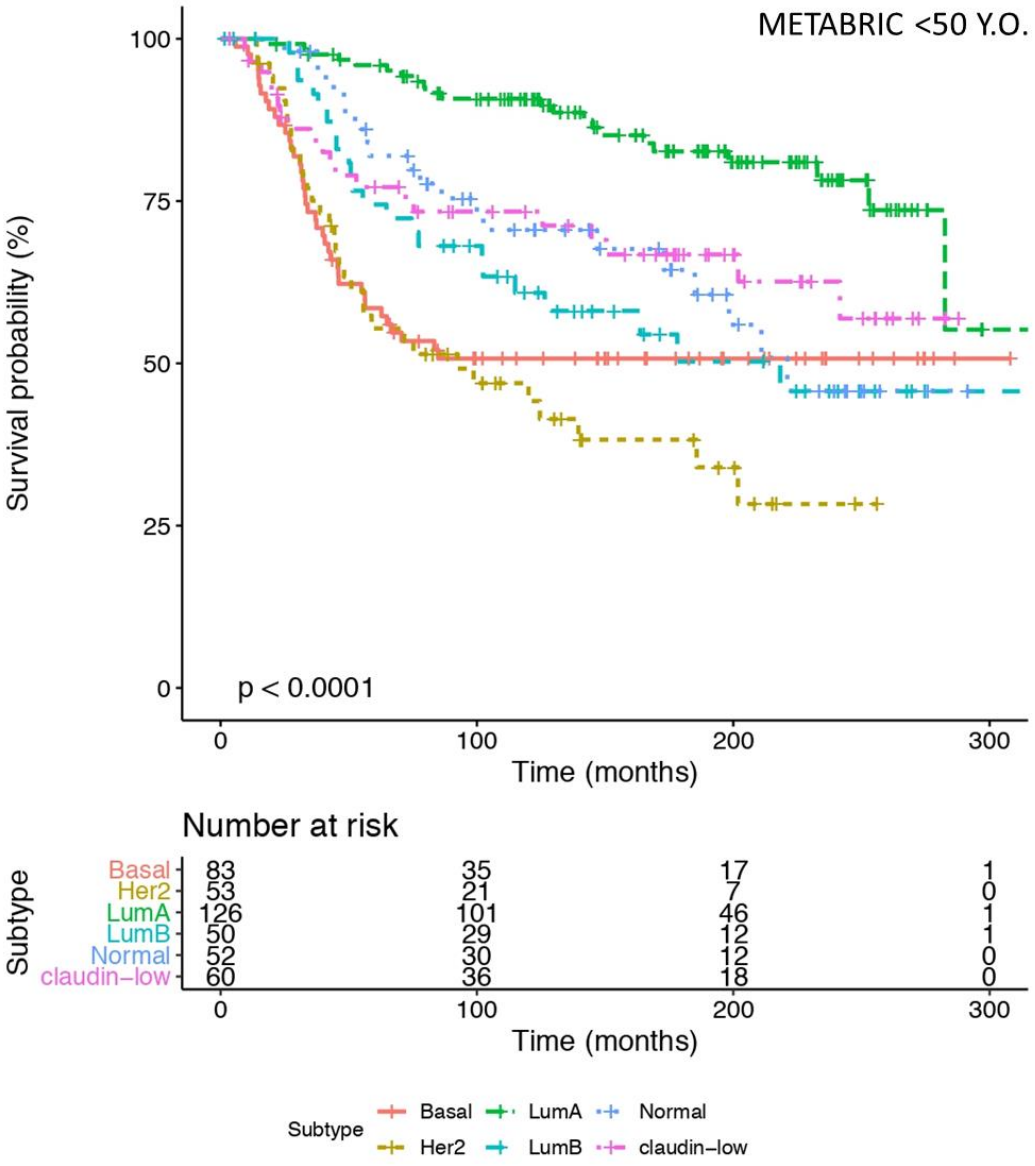

FIGURE S1 A. METABRIC survival by subtype classification in YWBC.

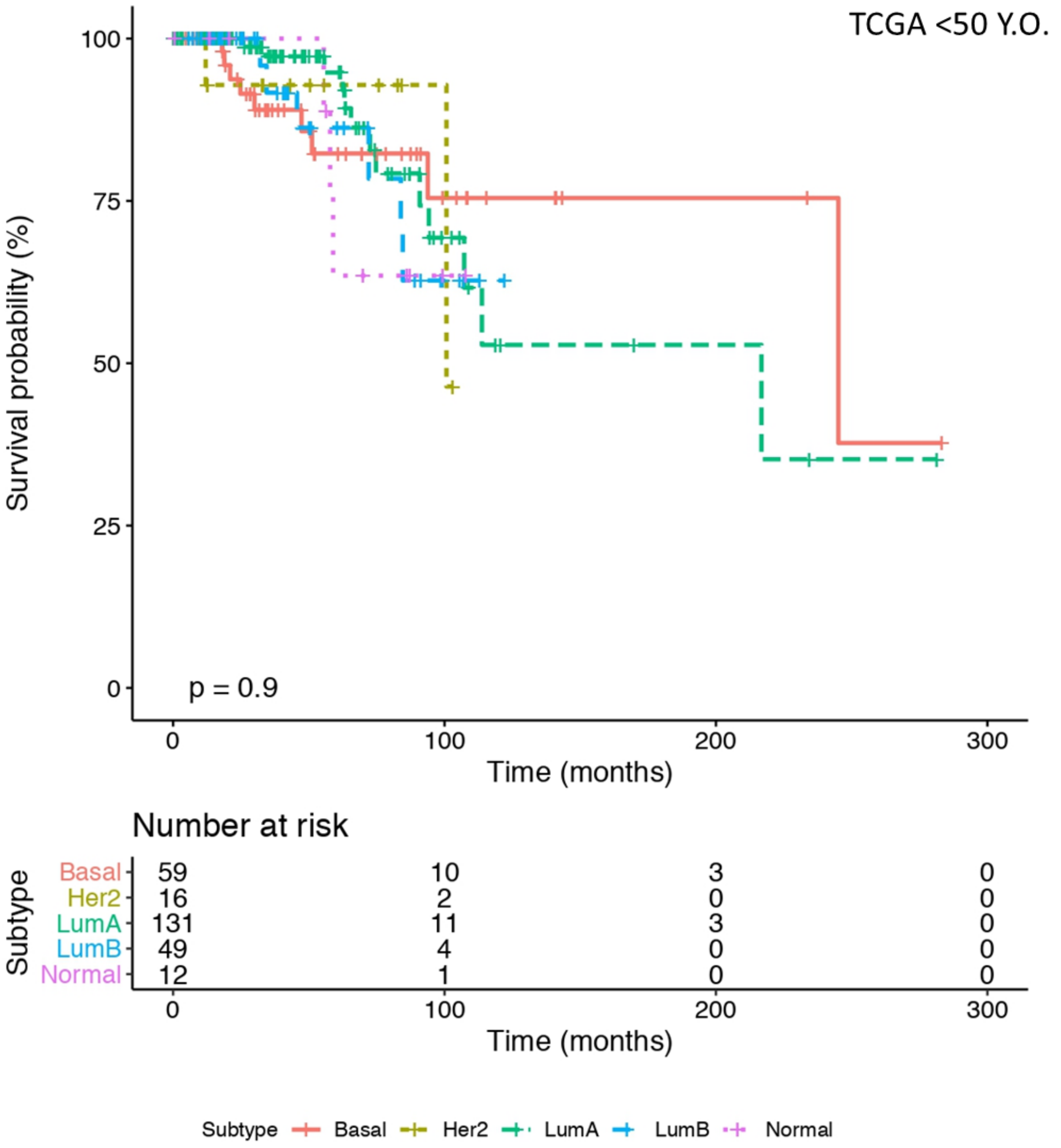

FIGURE S1 B. TCGA survival by subtype classification in YWBC.
